# Supplementary figures and images for: Antibacterial activity of graphene oxide nanosheet against multidrug resistant superbugs isolated from infected patients
Source: R Soc Open Sci. 2020 Jul 15;7(7):200640. doi: 10.1098/rsos.200640 (PMC7428267; doi:10.1098/rsos.200640)

| 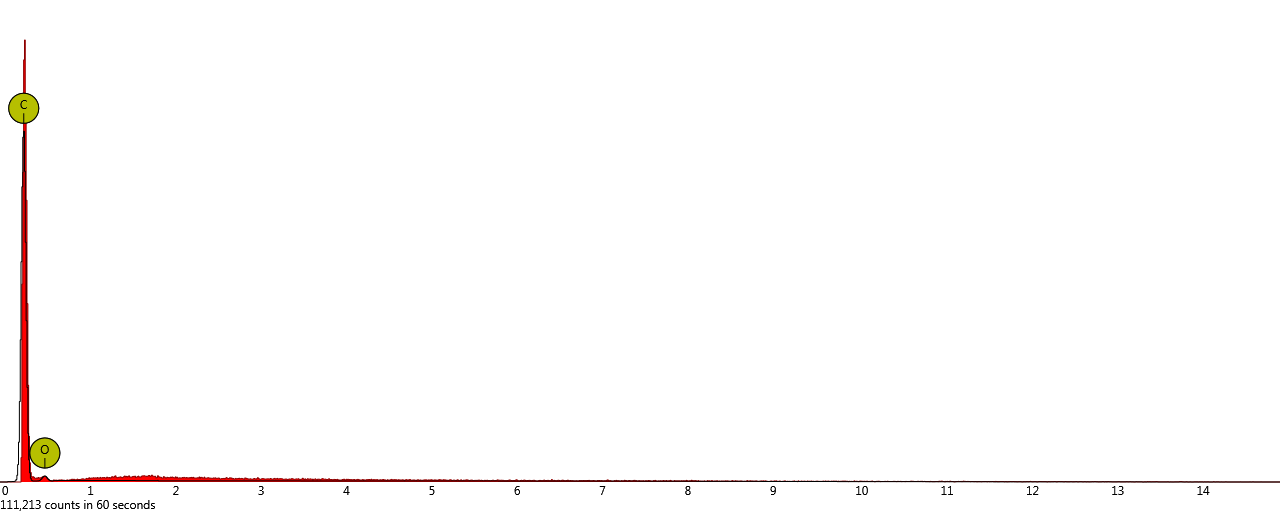  (a) |
| --- |
| 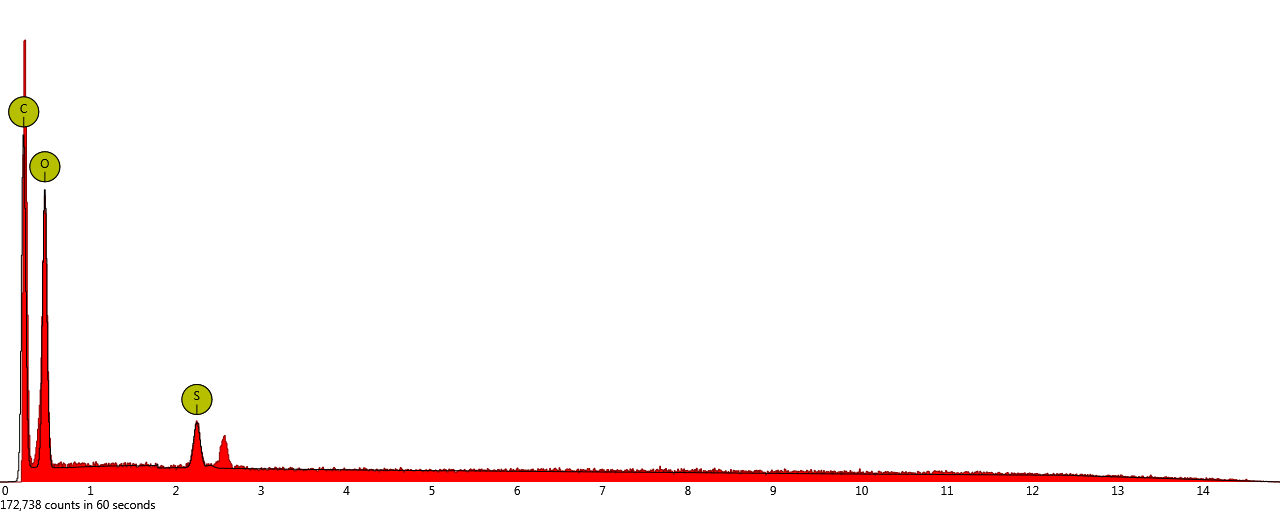  (b) |

**Supplementary Figure S1**

Supplement: Supplementary Figure S1 [file rsos200640supp1.doc]

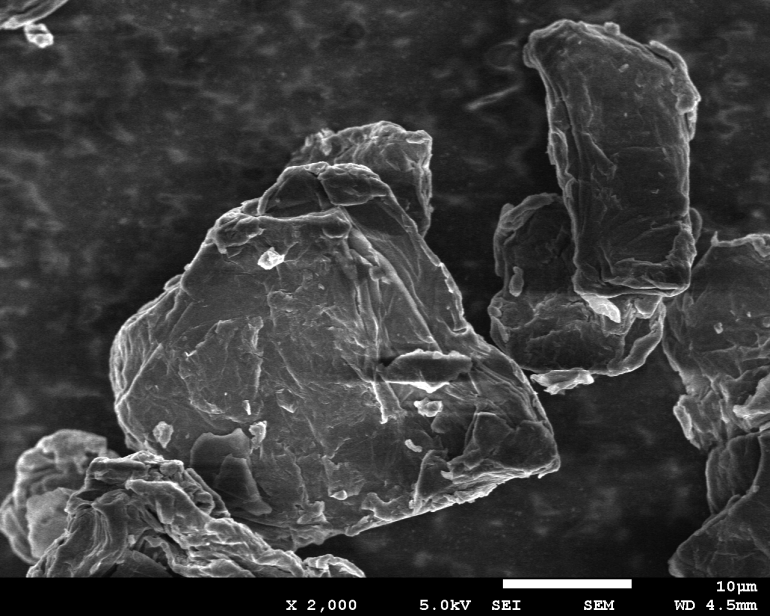

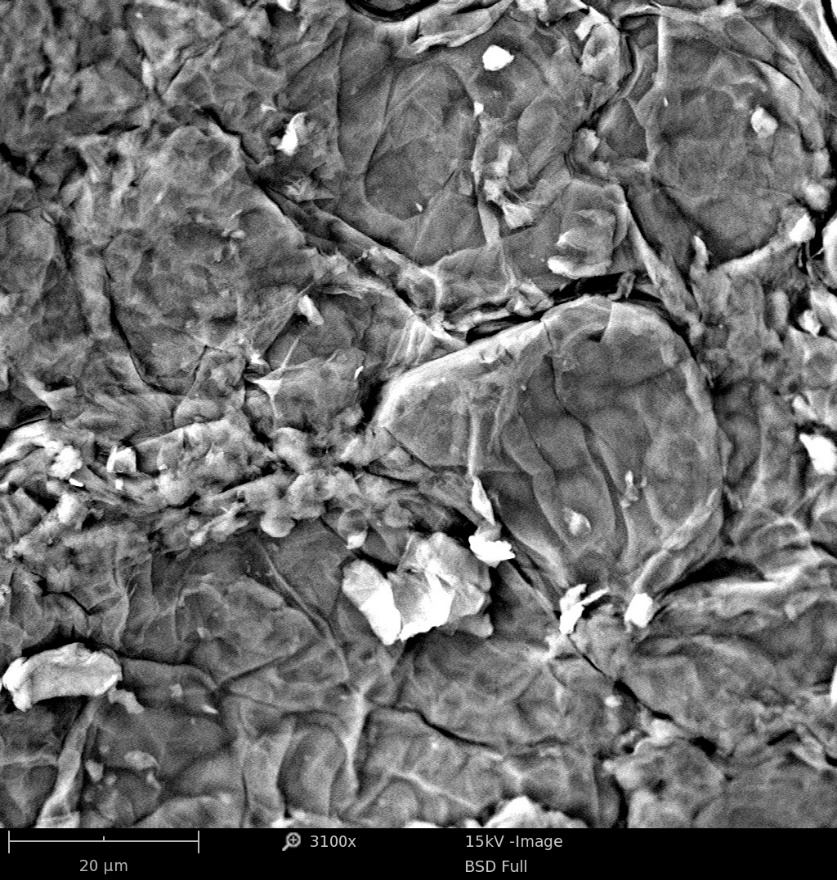


**(a)**

**(b)**

**10 µm**

**20 µm**

**Supplementary Figure S2**

Supplement: Supplementary Figure S2 [file rsos200640supp2.doc]

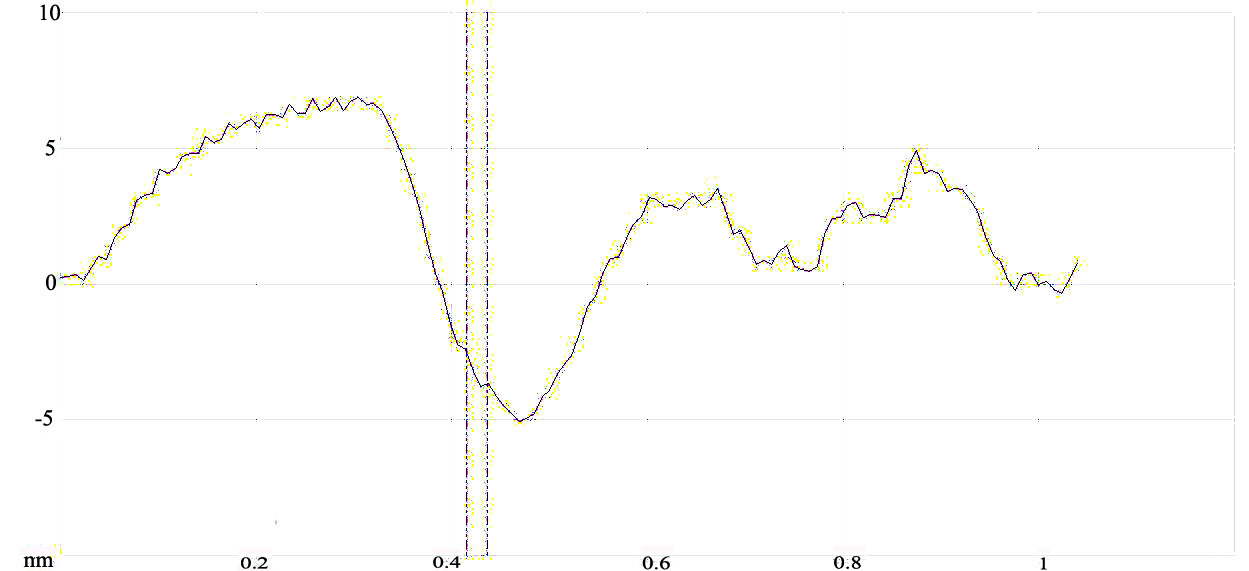

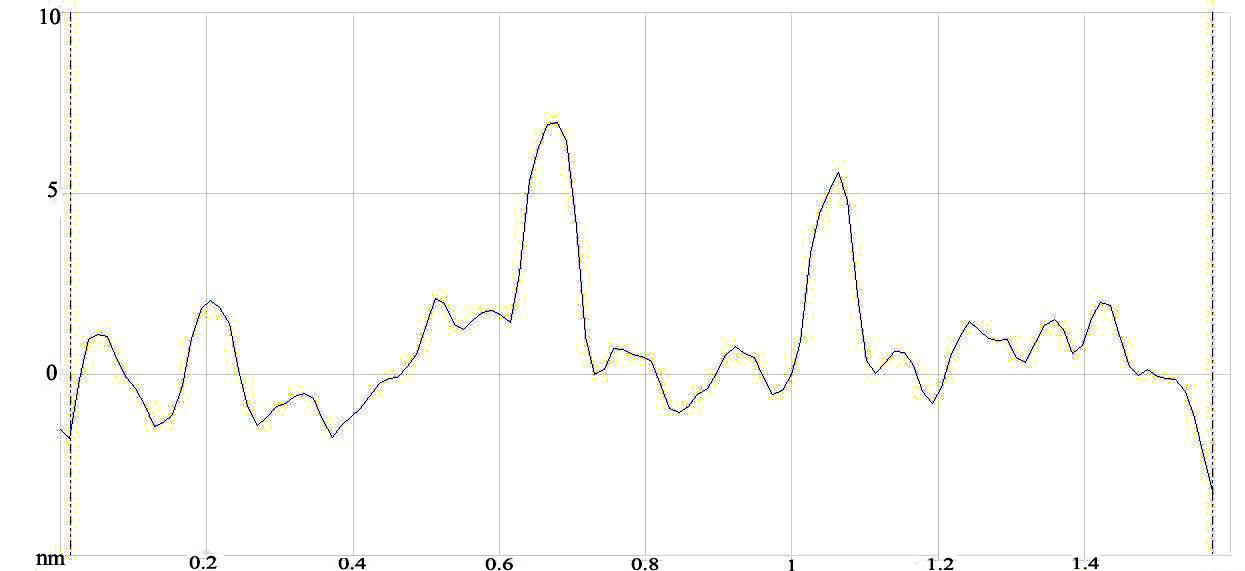


**Supplementary Figure S3**

**µm**

Supplement: Supplementary Figure S3 [file rsos200640supp3.doc]
