# Supplementary material for: Antibacterial activity of graphene oxide nanosheet against multidrug resistant superbugs isolated from infected patients
Source: R Soc Open Sci. 2020 Jul 15;7(7):200640. doi: 10.1098/rsos.200640 (PMC7428267; doi:10.1098/rsos.200640)
Supplement: Supplementary Table S1 [file rsos200640supp4.doc]

**Table S1. MIC and MBC of GO**

| | Isolates | Absence of blood | | Presence of blood | | | --- | --- | --- | --- | --- | | MIC | MBC | MIC | MBC | | *E. coli* | 0.065 | 0.13 | 0.065 | 0.13 | | *K. pneumoniae* | 0.065 | 0.13 | 0.065 | 0.13 | | *S. aureus* | 0.065 | 0.13 | 0.065 | 0.13 | | *P. aeruginosa* | 0.032 | 0.065 | 0.032 | 0.065 | | *P. mirabilis* | 0.065 | 0.13 | 0.065 | 0.13 | | *S. mercescens* | 0.032 | 0.065 | 0.032 | 0.065 | |
| --- | --- | --- | --- | --- | --- | --- | --- | --- | --- | --- | --- | --- | --- | --- | --- | --- | --- | --- | --- | --- | --- | --- | --- | --- | --- | --- | --- | --- | --- | --- | --- | --- | --- | --- | --- | --- | --- | --- | --- |
